# Spinal Schwannoma with Intradural Intramedullary Hemorrhage

**DOI:** 10.7759/cureus.1082

**Published:** 2017-03-06

**Authors:** Muhammad Nadeem, Salman Mansoor, Salman Assad, Fariha Ilyas, Ahmed H Qavi, Shoab Saadat

**Affiliations:** 1 MBBS, FCPS, Department of Urology, Pakistan Institute of Medical Sciences, Islamabad, Pakistan; 2 Department of Neurology, Shifa International Hospital, Islamabad, Pakistan; 3 Department of Medicine, Shifa Tameer-e-Millat University, Islamabad, Pakistan; 4 Department of Medicine, University of Texas at Austin, Dell Medical School, Austin, TX, USA; 5 Department of Medicine, Montefiore New Rochelle Hospital, Albert Einstein College of Medicine, New Rochelle, NY, USA; 6 Department of Nephrology, Shifa International Hospital, Islamabad, Pakistan

**Keywords:** sah, intradural, spine, schwannoma

## Abstract

Patients with spinal abnormalities infrequently present with intradural intramedullary bleeding. The more common causes include spinal trauma, arteriovenous malformations and saccular aneurysms of spinal arteries. On occasion, spinal cord tumors either primary or metastatic may cause intramedullary bleed with ependymoma of the conus medullaris. Spinal nerve sheath tumors such as schwannomas only rarely cause intradural intramedullary bleed, especially in the absence of spinal cord or nerve root symptoms. We report a case of spinal intradural schwannoma presenting with acute onset of quadriparesis. Cerebral angiography studies were negative but magnetic resonance imaging (MRI) of the spine revealed a large hemorrhagic tumor in the thoracolumbar junction. However, we suggest that the patients with intradural intramedullary bleed should be evaluated for underlying spine disease.

## Introduction

Spinal schwannomas are slow growing spinal nerve sheath benign tumors. These are diagnosed by either imaging studies incidentally or after manifesting symptoms like a backache and progressive neurological deficit [1]. These tumors are seen commonly in intradural extramedullary location and in fourth to the sixth decade of life. They rarely present with the acute emergency [2]. We report a case of thoracolumbar intradural intramedullary schwannoma with acute hemorrhage in an adult male patient who had acute onset of quadriparesis. Informed consent was obtained from the patient for this study.

## Case presentation

A 68-year-old female was presented with back pain since last one day. She was in her usual state of health when around the day before she felt dizzy; as she got out of bed, fell on the floor, banging her back on the bed. She received an injury to her back and was bruised on her arms and other parts of the body. The patient did not lose consciousness. Soon after her fall, she developed numbness of her legs and was gradually unable to move. She had urinary incontinence since last day and night which required urgent catheterization when brought to the hospital. Her pain had now spread to the spine. On physical examination, the Glasgow Coma Scale (GCS) is 15/15, lower back pain, spinous process is tender and straight leg raise test (SLR) > 90. The deep tendon reflexes were decreased, paraplegia and loss of sphincters. Extremities and blood vessels were warm and well preferred.

**Figure 1:**
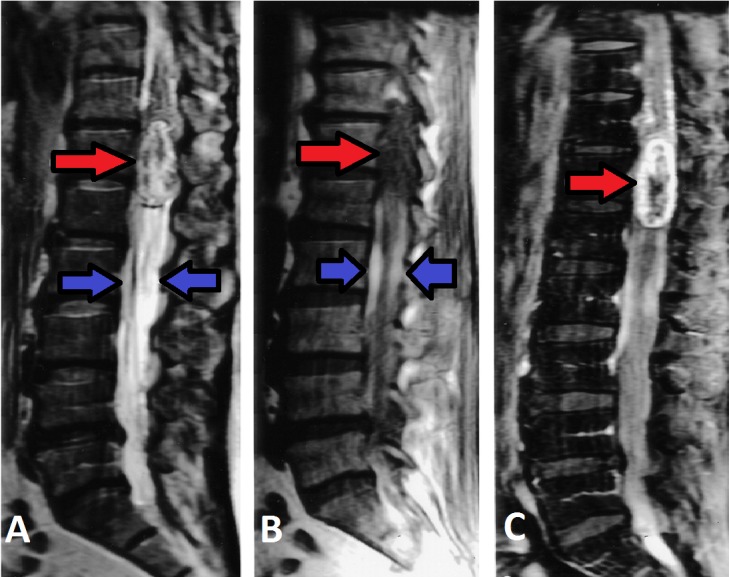
MRI of thoracolumbar spinal tumor Sagittal T2- [A] and T1-weighted [B] imaging shows a heterogeneous signal intensity of large mass at the conus medullaris (red arrows). The two hyperintense subdural hematomas located caudal to the mass (blue arrows). There is heterogeneous enhancement of the mass post-intravenus contrast [C]

**Figure 2:**
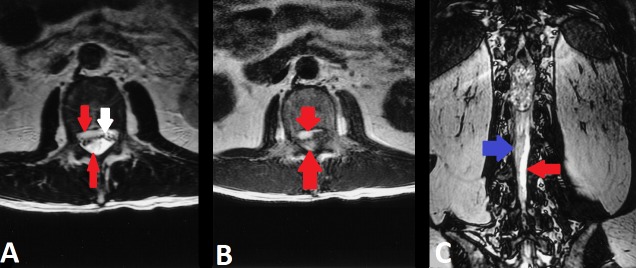
MRI spine Axial T2- [A] and T1-weighted [B] images show hyperintense subdural blood collections located both anterior and posterior (red arrows) to the cauda equina. The subdural blood is separate from the epidural fat signal intensity anteriorly [A, white arrow]. Coronal fast imaging with steady-state acquisition [C] shows the posterior blood collection (blue arrow) that is distinctly isolated from the hyperintense cerebrospinal fluid (red arrow)

Biochemical laboratory studies were unremarkable except for a mild decrease in levels of sodium (132 mEq/L), potassium (3.3 mEq/L) and chloride (92 mEq/L). MRI T1-weighted images show a large mass at the conus medullaris with heterogeneous signal intensity (Figure 1). MRI spine shows hyperintense subdural blood collections located both anterior and posterior to the cauda equina (Figure 2).

### Intra-operative Course

**Figure 3:**
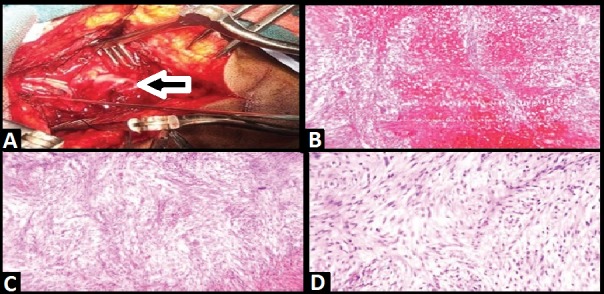
Operative and histopathology findings [A] The intraoperative picture shows the schwannoma with intratumoral hemorrhage (arrow). [B] Histopathological study of the mass shows macro hemorrhage within the mass [C, D] cellular area and loose myxoid area

The patient was offered surgery for exploration and spinal decompression. Coagulation parameters at the time of presentation were within normal limits. She underwent a thoracic laminectomy, exploration of the subdural space, evacuation of a compressive intradural intramedullary hematoma and resection of thoracic schwannoma. Upon exploration of the intradural space, a thick area of the compressive clot was noted, along with a traversing nerve root noted to have a reddish-tan colored small growth adherent to the clot, concerning for a nerve sheath tumor. The clot was evacuated, the tumor was resected. The specimen was sent for histopathology, post-operatively that revealed schwannoma [Figure 3].

At the time of discharge from the hospital to a rehabilitation facility, the patient had regained some function in the lower extremities (one-two out of five on neurological power exam) though she did not show any antigravity function on examination. There were many sessions done for physiotherapy involving venous pumps, therapeutic limp physio, and positioning.

## Discussion

Primary spinal cord tumors are classified based on their locations as extradural, intramedullary and intradural extramedullary. Surgical resection offers adequate treatment for intradural extramedullary tumors that include schwannomas, neurofibromas, and meningiomas [3]. Schwannomas are confined to the peripheral nerves or spinal nerve roots with well-defined borders without malignant transformation [4]. Various theories based on tumor locations and histologic features have been presented to scientific society to explain this process [5,7]. According to the vascular theory, spontaneous thrombosis of ectatic and hyalinized vessels of the tumor may occur leading to distal tumor necrosis and hemorrhage [6]. The mechanical theory, on the other hand, suggests that disturbance at the border of the tumor and the nerve tissue directs the hemorrhage into subarachnoid space [8-9]. Traction on vascular attachments to nerve roots may also cause interruption of the blood vessels on the tumor’s surface [10]. However, other probable reasons for the hemorrhage propensity in such large tumors of schwannomas can be explained due to central ischemic necrosis associated with both malignant transformation and neovascularization [10]. There is no definite histopathological evidence of malignant transformation in the present case, despite the history of trauma. Blood vessels were warm and well perfused. The patient was in her usual state of consciousness. Mechanical theory suggests subarachnoid hemorrhage in contrasts to our case of intradural intramedullary bleed [3]. We are of the view that rupture of the weak, ectatic tumor vessels and ensuing dissection of the blood into the subarachnoid space was caused by the degenerative changes accompanied with the mechanical stress at the conus.

## Conclusions

It is relatively rare for patients with spinal pathologies to present with intradural intramedullary bleed. This report of thoracolumbar schwannoma presenting clinically with intradural and intramedullary bleed illustrates the diagnostic difficulties for both neuroradiologists and neurosurgeons. It highlights the importance of an in-depth systemic review and a high index of clinical suspicion for spinal disease.
